# Factors associated with early antenatal care attendance among women in Papua New Guinea: a population‐based cross‐sectional study

**DOI:** 10.1186/s13690-021-00592-6

**Published:** 2021-05-06

**Authors:** Abdul-Aziz Seidu

**Affiliations:** 1 Department of Population and Health, College of Humanities and Legal Studies, University of Cape Coast, Cape Coast, Ghana; 2 College of Public Health, Medical and Veterinary Sciences, James Cook University, Townsville, Queensland Australia

**Keywords:** Antenatal care, Initiation, Papua New Guinea, Public Health, Pregnant women, Timing, Utilisation

## Abstract

**Background:**

Early initiation of antenatal care (ANC) is a key component of antenatal care, as suggested by the World Health Organisation (WHO). It helps in early identification and mitigation of adverse pregnancy-related complications. Despite this, a greater proportion of women worldwide still do not adhere to this recommendation. This study, therefore, sought to assess the prevalence and factors associated with early initiation of ANC among women in Papua New Guinea (PNG).

**Methods:**

A population-based cross-sectional study was conducted among 4,274 women using data from the 2016–2018 PNG Demographic and Health Survey (PDHS). The outcome variable was early initiation of ANC. Bivariate (chi-square) and multivariable logistic regression analyses were done and statistical significance was set at *p* < 0.05.

**Results:**

The prevalence of early ANC initiation was 23.0 % (CI = 20.8–24.6). The binary logistic regression analysis showed that working women had higher odds of early ANC attendance compared with those who were not working [AOR = 1.37, 95 %CI = 1.17 = 1.60]. The results also showed that women from Islands region had lower odds [AOR = 0.50, 95 %CI = 0.40–0.62] of early ANC attendance compared with those from Southern region. Finally, women with parity 3 had lower odds of early ANC attendance compared to those with parity 1[AOR = 0.64,95 % CI = 0.49–0.84].

**Conclusions:**

This study found a relatively low prevalence of early ANC uptake among women in PNG. The factors associated with early ANC attendance were region of residence, parity, and working status of mothers. To increase early ANC uptake, these factors should be considered when designing new policies or reviewing policies and strategies on ANC uptake to help increase ANC attendance, which can help in the reduction of maternal mortality.

## Background

Globally, maternal mortality is one of the major public health concerns [1]. As a result, it featured in the past Millennium Development Goals (MDGs) and well featured in its successor, Sustainable Development Goals (SDG) target 3.1. Antenatal care during pregnancy is one of the measures to reduce maternal mortality [2]. Gebresilassie et al. [3] defined first timing of antenatal care (ANC) attendance “*as the first-time pregnant women come to antenatal clinics to get care from health care professionals”.* During this period, health providers can determine the health status of the mother and the foetus. The WHO [4] recommends that pregnant women in low- and middle-income countries (LMICs) get at least four ANC visits and initiate early ANC follow-ups. In the light of the evidence-based benefits of ANC, in 2016, the WHO recommended eight visits for women in low- and middle-income countries, rather than the four recommended previously [5]. The 1st ANC timing should be before 16 weeks of gestational age (early ANC attendance or initiation), the 2nd visit between 24 and 28 weeks of gestation age, the 3rd visit between 30 and 32 weeks gestational age, and the 4th visit between 36 and 38 weeks of gestational age. This early initiation of ANC is critical to aid in early detection of pregnancy-related problems and adverse pregnancy outcomes, including low birth weight, still birth, intra uterine foetal death, and other complications [3–6].

Worldwide, approximately 830 and more than 303,000 women die each day and annually respectively due to pregnancy and childbirth-related complications [1]. From these numbers, the greater percentage (99 %) occur in LMICs [1, 7]. Papua New Guinea (PNG) is one of the Asian Pacific countries with the highest maternal mortality ratios [8, 9] and the major causes of these deaths are obstetric haemorrhage, sepsis, embolism, eclampsia and unsafe abortion [8]. Some of these complications can be detected through early initiation of ANC. Despite this, in many LMICs, women do not attend ANC at all or start seeking ANC late during pregnancy [3, 10], with global prevalence of 58.6 % (48.1 % in developing regions and 84.8 % in developed regions as well as 81.9 % in high-income countries and 24.0 % in low-income countries) [11].

Evidence suggests that age [6, 12, 13], wealth status [6, 14], work or employment [12, 15], level of education of women and their partners [3, 6, 12–14], marital status [12], place and region of residence [16], parity [17], pregnancy intentions [3, 12–15, 17], sex of household head, exposure to mass media [18], decision maker on healthcare, permission before seeking healthcare, money to seek healthcare, and distance to health facility [17] are associated with early ANC. These studies conducted in various parts of the world have consistently found a low level of early ANC initiation. Despite this evidence, to the best of my knowledge, none of such studies have been conducted in PNG using nationally representative dataset to determine the prevalence and factors associated with early initiation of ANC. The findings of such a national study would be critical because they will aid in identifying particular women to target in order to increase the use of ANC services, which can help reduce maternal mortality in PNG.

## Materials and methods

### Data and sampling design

The data used for this study forms part of the 2016–2018 PDHS, which was collected from October 2016 to December 2018. The survey adopted a two-stage stratified sampling technique. Each province was stratified into urban and rural areas, yielding 43 sampling strata, with the exception of National Capital District, which had no rural area. Samples of census units (CUs) were selected independently in each stratum in two stages. In the first stage, 800 CUs were selected with probability proportional to CU size, which is the number of residential households found in the CU during the 2011 National Population and Housing Census (NPHC) [19]. Some of the selected clusters were large, with more than 200 households. To minimise the task of the listing team, these selected clusters were segmented. Only one segment was selected for the survey, with probability proportional to segment size. Household listing was conducted only in the selected segment. This means that a cluster is either a CU or a segment of a CU. In the second stage of selection, a fixed number of 24 households per cluster were selected with an equal probability systematic selection from the newly created household listing, resulting in a total sample size of approximately 19,200 households. All women aged 15–49 who were usual members of the selected households or who spent the night before the survey in the selected households were eligible for individual interview. A total of 17,505 households were selected for the sample, of which 16,754 were occupied and 16,021 were successfully interviewed (96 % response rate). In the interviewed households, 18,175 women age 15–49 were identified for individual interviews but 15,198 women were reached (84 % response rate). The final sample size for this study consists of 4,274 women who indicated they had ever attended ANC prior to the survey. Details of the methodology, pretesting, training of field workers, the sampling design, and selection are available in the PDHS final report [19] which is also available online at https://dhsprogram.com/publications/publication-fr364-dhs-final-reports.cfm.

### Derivation of study variables

#### Outcome variable

The outcome variable for this study is ANC attendance. It was derived it from the question “How many months pregnant were you when you first received antenatal care for this pregnancy?” The responses were in months. It was then dichotomised to early initiation of ANC= “1” if women reported attending at 3 months or earlier and Late initiation= “0”, after 3 months [6, 13, 15, 17, 18, 20].

## Independent variables

Eighteen independent variables were considered in this study. They were chosen based on two reasons, thus, their availability in the dataset [19] and conclusion drawn on them to be associated with ANC attendance in previous studies [6, 13, 15, 17, 18, 20]. The variables comprised maternal age, wealth, working status, education, partner’s education, marital status, place of residence, region of residence, parity, pregnancy intention, permission to go to hospital, getting money for treatment, distance to health facility, frequency of listening to radio, frequency of watching television, frequency of reading newspaper or magazine, and sex of household head. Some of these variables were recoded for meaningful, easy interpretation of results. Parity (birth order) was categorised as one birth= “1”, two births= “2”, three births= “3” and four births or more= “4”. Occupation was captured as working= “1” and not working= “0” and decision maker on healthcare was captured as alone= “1” and not alone= “2” (see Table 1).

**Table 1:** Background characteristics and early ANC attendance among women in PNG

Variable	***N***=4,274		Early ANC attendance	
	Frequency	Percentage	No (%) 3306(77.0%)	Yes (%) 968(23%)
**Age (*χ***^***2***^ **=13.3,*p*=0.038)**				
15-19	156	3.6	77.6	22.5
20-24	969	22.7	75.2	24.8
25-29	1,156	27.0	78.9	21.1
30-34	906	21.2	81.7	18.3
35-39	658	15.4	78.5	21.5
40-44	340	8.0	79.5	20.5
45-49	89	2.1	83.9	16.1
**Wealth (*χ***^***2***^ **=14.0,*p*=0.007)**				
Poorest	641	15.0	74.7	25.4
Poorer	787	18.4	76.0	24.0
Middle	881	20.6	78.5	21.5
Richer	947	22.2	81.6	18.4
Richest	1,018	23.8	79.6	20.4
***Working*** **(*χ***^***2***^ **=9.1,** ***p*=0.003)**				
Not working	2,746	64.3	80.3	19.7
Working	1,528	35.7	76.5	23.5
**Education (*χ***^***2***^ **=16.9,** ***p*=0.001)**				
No education	757	17.7	74.2	25.9
Primary	2,189	51.2	80.0	20.0
Secondary	1091	25.5	80.1	19.9
Higher	237	5.5	72.3	27.8
**Partner Education (*χ***^***2***^ **=7.30,** ***p*=0.063)**				
No education	663	15.5	75.8	24.2
Primary	1,774	41.5	79.5	20.5
Secondary	1,396	32.7	80.0	20.1
Higher	441	10.3	75.6	24.4
**Marital status (*χ***^***2***^ **=0.83,*p*=0.362)**				
Married	3,570	83.5	78.6	21.4
Cohabiting	704	16.5	80.1	19.9
**Region (*χ***^***2***^ **=66.7,** ***p*<0.001)**				
Southern region	922	21.6	76.7	23.3
Highlands region	1,579	37.0	73.4	26.7
Momase region	1,082	25.3	78.1	21.9
Islands region	690	16.2	86.9	13.1
**Residence (*χ***^***2***^ **=1.2,** ***p*=0.278)**				
Urban	555	13.0	80.0	20.1
Rural	3,719	87.0	78.4	21.6
**Parity (*χ***^***2***^ **=20.0,** ***p*<0.001)**				
1	1,034	24.2	75.3	24.7
2	903	21.1	76.7	23.4
3	781	18.3	83.0	17.0
4+	1,556	36.4	80.2	19.8
**Pregnancy intention (*χ***^***2***^ **=1.4,** ***p*=0.490)**				
Planned	3,001	70.2	79.3	20.8
Mistimed	526	12.3	77.0	23.0
Unwanted	747	17.5	78.4	21.6
**Permission to go to hospital (*χ***^***2***^ **=0.6,** ***p*=0.453)**				
Big problem	1,210	28.3	79.6	20.4
Not a big problem	3,064	71.7	78.5	21.5
**Getting money needed for treatment (*χ***^***2***^ **=2.10,** ***p*=0.147)**				
Big problem	2,521	59.0	78.0	22.0
Not a big problem	1,753	41.0	79.9	20.1
**Distance to health facility (*χ***^***2***^ **=2.44,** ***p*=0.118)**				
Big problem	2,275	53.2	77.8	22.2
Not a big problem	1,999	46.8	79.8	20.2
**Decision maker on healthcare (*χ***^***2***^ **=0.0,*p*=1.00)**				
Not alone	2,996	70.1	78.8	21.2
Alone	1,278	29.9	78.8	21.2
**Frequency of reading newspaper or magazine (*χ***^***2***^ **=7.4,** ***p*=0.025)**				
Not at all	2,566	60.0	77.8	22.2
Less than once a week	930	21.8	81.9	18.1
At least once a week	778	18.2	78.1	21.9
**Frequency of listening to radio (*χ***^***2***^ **=4.5,** ***p*=0.106)**				
Not at all	2,622	61.3	79.3	20.7
Less than once a week	870	20.4	80.0	20.0
At least once a week	782	18.3	76.2	23.8
**Frequency of watching television (*χ***^***2***^ **=1.8,** ***p*=0.413)**				
Not at all	3,188	74.6	79.3	20.7
Less than once a week	439	10.3	77.8	22.2
At least once a week	647	15.1	77.3	22.8
**Sex of household head (*χ***^***2***^ **=0.2,** ***p*=0.670)**				
Male	3,669	85.8	78.7	21.3
Female	605	14.2	79.5	20.5

Source: 2016-18 PNG DHS

### Statistical analyses

Descriptive, bivariate and multiple logistic regression analyses were conducted. The descriptive analysis (frequencies and percentages) was used to describe the study sample. The bivariate analysis was conducted using Chi-square test to assess the differentials in the prevalence of early ANC attendance across all the independent variables. All the variables that appeared statistically significant (*p* < 0.05) were moved to the multiple logistic regression analysis stage. The results for the multiple logistic regression analysis were presented as adjusted odds ratios (AOR) along with their respective 95 % confidence intervals (CIs) signifying precision. Using the variance inflation factor (VIF), I found that the multicollinearity test showed no evidence of collinearity among the independent variables (Mean VIF = 1.4, Max VIF = 1.72, Minimum = 1.01**)**. The sample weight (wt) was used to account for the complex survey (svy) design and generalizability of the findings. All the analyses were done with Stata version 14.2 for MacOS.

### Ethical issues

The 2016–2018 PDHS report indicated that ethical approval was granted by the ICF Institutional Review Board [18]. Both written and verbal informed consent were also sought from all the participants during the data collection exercise. The dataset was requested on 10th March, 2020.The dataset can be accessed at: https://dhsprogram.com/data/dataset/Papua-New-Guinea_Standard-DHS_2017.cfm?flag=0.

## Results

Table 1 presents the prevalence of early ANC attendance among women who sought ANC at PNG. It was found that less than a quarter (23 %) had initiated ANC attendance early. Table 1 also shows the background characteristics of the women. It was found that 27 % were aged 25–29. Approximately 23 % were in the richest wealth category and 64.3 % were not working. Slightly more than half (51.2 %) have attained primary level of education. The majority (83.5 %) were married. The majority (87.0 %) were also in rural areas while 36.4 % had 4 or more children. It was also found that 24.8 % of the women aged 20–24 had initiated ANC attendance within the first trimester. It was also found that 25.4 % of the poorest, 23.5 % of those working, 25.9 % of those with no formal education and 21.4 % of those who were married initiated their ANC early (see Table 1).

### Determinants of early ANC attendance among women in PNG

Table 2 presents the logistic regression analysis of determinants of early ANC attendance among women in PNG. It was found that working women had about 1.37 higher odds of early ANC attendance compared with those who were not working [AOR = 1.37, 95 %CI = 1.17 = 1.60]. The results also showed that women from Islands region had 0.50 lower odds [AOR = 0.50, 95 %CI = 0.40–0.62] of early ANC attendance compared with those from Southern region. Finally, women with parity 3 had 0.64 lower odds of early ANC attendance compared to those with parity 1 [AOR = 0.64,95 % CI = 0.49–0.84].

**Table 2:** Logistic regression analysis of determinants of early ANC attendance in PNG

Variable		[95% CI]		***P***-value
	AOR	Lower Bound	Upper Bound	
**Age**				
15-19	Ref	Ref	Ref	Ref
20-24	1.26	0.83	1.92	0.282
25-29	1.12	0.73	1.73	0.603
30-34	1.00	0.63	1.58	0.994
35-39	1.25	0.77	2.02	0.366
40-44	1.18	0.70	1.99	0.529
45-49	0.82	0.39	1.70	0.585
**Wealth status**				
Poorest	Ref	Ref	Ref	Ref
Poorer	1.01	0.77	1.33	0.935
Middle	0.97	0.74	1.28	0.847
Richer	0.88	0.66	1.15	0.344
Richest	0.81	0.60	1.08	0.143
**Working status**				
Not working	Ref	Ref	Ref	Ref
**Working**	**1.37**	**1.17**	**1.60**	**<0.001**
**Educational level**				
No formal education	Ref	Ref	Ref	Ref
Primary	0.86	0.68	1.08	0.194
Secondary	0.91	0.69	1.21	0.531
Higher	1.26	0.83	1.91	0.278
**Region**				
Southern region	Ref	Ref	Ref	Ref
Highlands region	1.14	0.93	1.39	0.202
Momase region	0.91	0.74	1.13	0.41
**Islands region**	**0.50**	**0.40**	**0.62**	**<0.001**
**Parity**				
1	Ref	Ref	Ref	Ref
2	0.96	0.76	1.20	0.692
**3**	**0.64**	**0.49**	**0.84**	**0.001**
4+	0.79	0.61	1.04	0.088
**Frequency if reading Newspaper/Magazine**				
Not at all	Ref	Ref	Ref	Ref
Less than once a week	0.83	0.67	1.02	0.072
At least once a week	0.99	0.79	1.26	0.961

Source: 2016-18 PDHS

Exponentiated coefficients; 95% confidence intervals [CIs] in square brackets

*ref* reference, *AOR* Adjusted Odds Ratios

*Significant results in bold

## Discussion

This study sought to assess the prevalence and determinants of early initiation of ANC attendance among women in PNG. The WHO [4] recommends that every woman should make an effort to attend ANC within the first trimester due to the fact that it is one of the critical stages during pregnancy. Despite this recommendation, a greater number of women in most LMICs do not adhere to this recommendation [6, 20]. In this current study, less than a quarter (23 %) of women in PNG initiated ANC attendance in their first trimester during pregnancy. This is similar in several LMICs including Ethiopia (21.7 %) [17], (27.5 %) [3, 21], (26.2 %) [21]; Benin (24.6 %) [22]; Tanzania (29 %) [23] and Uganda (27.9 %) [24]. The prevalence of early ANC initiation in this study is, however, higher than what was found in other studies done in Zambia (17 %) [25], Nigeria (15.4 %) [26], Tanzania (12.4 %) [27], and Ethiopia (17.4 %) [20], (13.2 %) [28].

Nonetheless, the prevalence recorded in this study is lower than the global prevalence of 58.6 % [11] as well as several other studies elsewhere showing prevalence between 32.7 and 72 % [6, 12, 14–17, 29–32]. The differences in the study findings could be explained by the differences in study settings. This lower proportion of early ANC uptake in this study implies that most women in PNG may not enjoy full importance associated with early ANC uptake [6, 11]. Some of these benefits include precautionary measures, timely disease detection and treatment strategies, including use of iron and folate supplements for the treatment of anaemia, Intermittent Preventive Treatment for malaria in pregnancy, immunisation against tetanus and Tuberculosis, and detection of Sexually Transmitted Infections including HIV and AIDS to prevent mother to child transmission as well as health education in general including appropriate nutrition and personal hygiene [6, 11].

Again, working women had higher odds of early ANC attendance compared to women who were not working. This is consistent with several empirical studies in various parts of the world such as Ghana [33], Nepal [34], Ethiopia [35, 36], and Nigeria [37, 38]. However, the results are different from the finding in the South African studies by Ebonwu et al. [11] and Solarin and Black [39] which showed that working women present late for their first ANC. The probable explanation they offered was that employed women were busy compared to those who are not working and as a result attend ANC in later part of their pregnancy duration.

It was also found that women in PNG with parity three had lower odds of early ANC initiation compared to those with parity one. This is in line with several previous studies [40–44] on the association between parity and ANC uptake, which consistently indicates that high parity is associated with late initiation and low uptake of antenatal care services. There are various possible pathways that studies have reported to show this association. For example, Dangal [45] has reported that successive pregnancies might carry lower risks for complications if the first pregnancy and birth were uncomplicated. Pallikadavath, Foss and Stones [46] have also indicated that women who do not experience any complication for a previous pregnancy might not see the need to seek early ANC during their current pregnancy. In addition, Simkhada et al. [42] and Pallikadavath, Foss and Stones [46] explained that some women with other children might be occupied with the responsibilities of taking care of them and might find it difficult to attend early ANC. Pell et al. [47] are also of the view that high parity women who have had previous successful pregnancies might think they are well ‘experienced’ and now used to the routine care offered during ANC and so might delay ANC initiation. In addition, Simkhada et al. [42] also stated that insufficient resources and deleterious experiences with ANC providers during earlier pregnancies might make women hesitant to seek early ANC in current pregnancies. The study also found that regional variations exist in the likelihood of early ANC attendance. Specifically, women in the Islands region had lower odds of early ANC attendance compared to those in the Southern regions. This is also consistent with previous studies which found that variations exist in the various regions and the uptake of antenatal care services [48–51]. The possible reasons for this could be differences in access to healthcare facilities among the regions and the level of socio-economic development.

### Strength and limitation of the study

The study is fraught with certain limitations that demand acknowledging. First, the study design makes it impossible to draw causal interpretation on the findings obtained. Second, since the study demanded women to recall previous events, there is the possibility of social desirability and recall biases. Aside from these, the relatively large sample size and the use of nationally representative dataset could make the findings generalisable to women in their reproductive age in PNG. The study also employed various techniques to collect accurate data and there was a relatively high response rate during the data collection. The study also did extensive literature search in order to control for several factors associated with early ANC attendance.

## Conclusions

This study found a relatively low prevalence of early ANC initiation among women in PNG. The factors associated with early ANC attendance were region of residence, parity, and working status of mothers. To increase early ANC uptake, these factors should be considered when designing new policies or reviewing policies and strategies on ANC uptake to help increase ANC attendance which can help in the reduction of maternal mortality.

## Data Availability

The dataset can be accessed at https://dhsprogram.com/data/dataset/.

## Abbreviations

AOR: Adjusted Odds Ratio
CI: Confidence Interval
VIF: Variance Inflation Factor
SDG: Sustainable Development Goal
ANC: Antenatal Care
WHO: World Health Organization
